# The Relationship between Parkinson Disease and Brain Tumor: A Meta-Analysis

**DOI:** 10.1371/journal.pone.0164388

**Published:** 2016-10-20

**Authors:** Rong Ye, Ting Shen, Yasi Jiang, Lingjia Xu, Xiaoli Si, Baorong Zhang

**Affiliations:** Department of Neurology, Second Affiliated Hospital, School of Medicine, Zhejiang University, Hangzhou, Zhejiang, China; Hokkaido Daigaku, JAPAN

## Abstract

**Objective:**

Epidemiological studies have investigated the association between Parkinson disease (PD) occurrence and the risk of brain tumors, while the results remain controversial. We performed a meta-analysis to clarify the exact relationship between PD and brain tumors.

**Methods:**

A systematic literature search was conducted using PubMed, Embase, ScienceDirect and CBM (China Biology Medicine Disc) before February 2016. Eligible studies were those that reported risk estimates of brain tumors among patients with PD or vice versa. A random-effects model was used to calculate the pooled odds ratio (OR) of the outcomes. Subgroup analyses and sensitivity analysis were conducted to explore the potential sources of heterogeneity.

**Results:**

In total, eight studies involving 329,276 participants met our inclusion criteria. The pooled OR was 1.51 (95%CI 1.21–1.89), indicating that PD carried a higher risk of brain tumor. Analyses by temporal relationship found that the occurrence of brain tumor was significantly higher after the diagnosis of PD (OR 1.55, 95% CI 1.18–2.05), but not statistically significant before PD diagnosis (OR 1.21, 95%CI 0.93–1.58). Subgroup analysis showed that gender differences, ethnicity differences and the characteristic of the tumor (benign or malignant) did not make much change in the association between brain tumor and PD.

**Conclusions:**

Our meta-analysis collecting epidemiological studies suggested a positive association of PD with brain tumors, while the influence of anti-parkinson drugs and ascertainment bias could not be excluded. Further studies with larger sample size and more strict inclusion criteria should be conducted in the future.

## Introduction

Parkinson disease (PD) is the second most common neurodegenerative disease, estimated to occur in approximately 1% of individuals older than 60 years, a number predicted to more than double by 2030 because of the aging population[1]. Resting tremor, rigidity, hypokinesia, and postural instability are considered as the four cardinal motor symptoms of PD resulting from the loss of dopaminergic neurons in the substantia nigra pars compacta[2]. However, the exact pathological mechanism of PD has not been well established and fully understood despite of the Braak staging[3, 4], causing it remaining incurable and irreversible[5]. Hence, countless efforts have been made to explore the potential physiopathologic mechanisms and to seek the possible therapeutic targets for PD.

In recent years, accumulating epidemiological and clinical studies have reported the relationship between PD and cancers, lighting the way to probe into the potential common pathogenic pathway involved in both diseases[6]. While most researches reveal that PD seems to protect against many forms of cancer, the term “inverse cancer comorbidity” has been used to describe the apparent protective anti-cancer effect of PD[7]. This general pattern has been also affirmed by a meta-analysis of 29 studies involving 107,598 PD patients, which found that PD was inversely related to most types of cancers, including both smoking-related and non-smoking-related cancers[8].

Nevertheless, different from the general pattern mentioned above, several recent studies have indicated that brain tumors have higher morbidity among patients with PD[9, 10], leading to the situation that results remain controversial among studies investigating the association between PD and brain tumors. Therefore, we conducted this systematic review and meta-analysis in order to provide a quantitative assessment of current epidemiologic evidence on brain tumors in relation to PD and explore the potential factors affecting the association between them.

## Methods

### Search strategy

All methods were pre-specified in a study protocol, which is available in S1 File. We performed this meta-analysis according to the recommendations of the Preferred Reporting Items for Systematic Reviews and Meta-analysis (PRISMA) (S1 Table) [11]. Articles before 28 February, 2016 were searched in four major electronic databases, including PubMed, Embase, ScienceDirect and CBM (China Biology Medicine Disc) by two independent investigators (R.Y. and T.S.). Relevant text words and medical subject headings about PD, brain tumor and brain cancer were included in the search strategy without restrictions. The search strategy for PubMed is detailed in S2 File. In addition, references of the retrieved articles were also screened to identify additional related studies.

### Eligibility criteria

The studies eligible for this meta-analysis had strictly meet the following criteria: (1) studies that investigated both PD and brain tumor; (2) studies that reported a measure of association (including an odds ratio [OR], relative risk [RR], or standardized incidence/event ratio [SIR/SER]), with 95% confidence intervals (CIs), for the association between PD and brain tumor; (3) studies that published in peer-reviewed journals without language restrictions; (4) studies that had a longitudinal design. As was described, all types of brain tumors, including both benign brain tumor and malignant brain cancer, were eligible in this study.

### Quality assessment and data extraction

The quality of cohort studies was appraised according to the 9-star Newcastle-Ottawa Quality Assessment Scale[12]. Two investigators (R.Y. and T.S.) independently rated the quality of the retrieved studies and extracted data from eligible studies including first authors, publication year, sample size, mean age, regions, follow-up time in years, type of study design, PD diagnosis time and adjusted risk estimates and their 95% CIs. Disagreements were solved by discussion or involvement a third reviewer if necessary.

### Statistical analysis

We managed all data using STATA 12.0 (Stata Corporation, College Station, TX, USA) and set odds ratio (OR) at a 95% confidence interval (95% CI) to assess the association between PD and brain tumor. As previously reported, the involved measures (OR, RR, HR or SIR) were treated equally under the assumption that both PD and brain tumors are rare. As ln odds ratios (lnORs) were considered to obey normal distribution, lnORs and the corresponding ln lower limits (lnLLs) and ln upper limits (lnULs) were used as data points in pooling analysis.

We estimated the between study heterogeneity across all eligible comparisons by performing the *X*^*2*^ tests (assessing the p-value) and the *I*^*2*^ statistic. Random-effects meta-analysis models were used to allow for between study heterogeneity. Sensitivity analyses were conducted so that the influence of each individual result could be assessed with the pooled data. Study design, PD diagnosis time, gender difference, characteristics of the tumor and ethnicity were used as stratifying variables in subgroup analysis. We also conducted the funnel plots, the Egger’s test[13] and the Begg’s test[14] to assess the potential publication bias if the included studies were more than 10 in each analysis.

## Results

### Eligible studies

**Fig 1:**
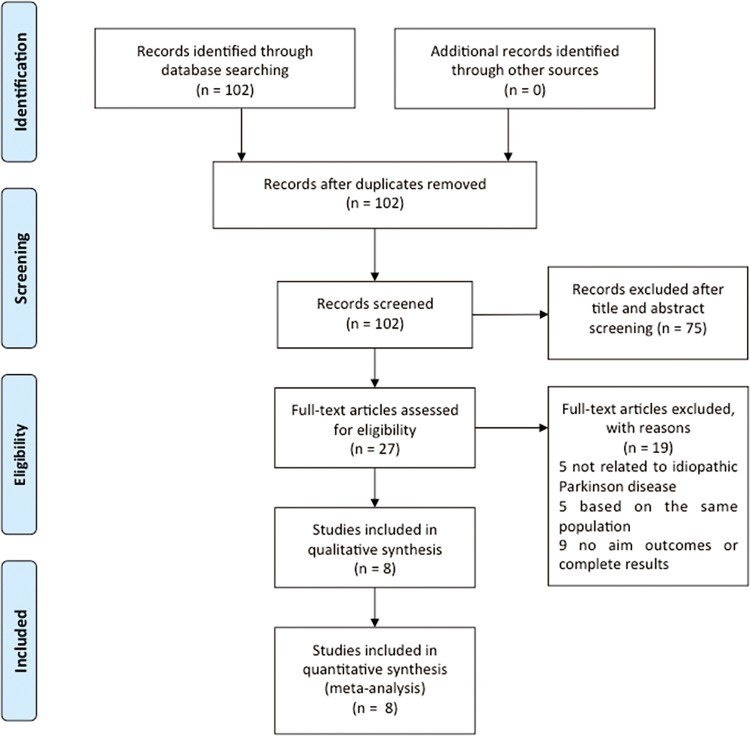
PRISMA flow chart of literature searches and results.

The electronic search identified 102 potentially relevant articles (Fig 1). After checking duplicates and carefully reviewing the titles and abstracts, we excluded 75 publications that failed to meet the inclusion criteria: 51 articles were excluded because of a lack of direct relevance, 5 were case reports, 8 were review articles, and 11 were meeting abstracts. Ultimately, 27 articles were identified with full text assessing, and 19 articles were subsequently excluded for the following reason: not relating to idiopathic Parkinson disease (n = 5), being based on the same population (n = 5), no available outcomes or complete results (n = 9). Eventually, a total of eight studies involving 329,276 participants were analyzed in this meta-analysis.

### Study characteristics

Characteristics of the 8 eligible studies included in the meta-analysis are presented in Table 1 [15–22]. Among the 8 studies involving 329,276 participants, 6 studies targeted at Caucasian and 2 studies were from Asia. Nearly all included eight studies were based on general population or community-based population except for Ong et al.[19], the data of which was from hospital. Besides, in the 8 studies on brain tumor and PD, 7 were cohort studies and one was case-control study. Only 1 of the 8 studies was explicitly designed to assess the relationship between brain tumor and PD, whereas others assessed brain tumor as part of their analyses on PD and other site-specific tumors or medical conditions of interest. Furthermore, in terms of the nature of brain tumors, 2 studies reported benign brain tumors, while others investigated the association between malignant brain cancers and PD. As participants were divided into different groups by PD diagnosis time and the nature of tumor in some original studies, three patient groups in study of Wirdefeldt et al.[18] and four patient groups in study of Fois et al.[22] were considered separately for pooling analysis, which contributed to a total of 13 groups for assessing the relationship between brain tumor and PD.

**Table 1:** Characteristics of Individual Study in the Meta-analysis.

Study	Year	Region	Study design	Sample size	Ethnicity	PD ascertainment	Brain tumor ascertainment	Brain tumor identification*	Characteristics of the tumor
Tang et al.[15]	2015	Taiwan	Cohort	2998	Asian	ICD-9	ICD-9	After	Benign/ Malignant
Lin et al.[17]	2015	Taiwan	Cohort	62023	Asian	ICD-9	ICD-9	After	Malignant
Wirdefeldt et al.	2014	Sweden	Cohort	11786	Caucasian	ICD-7, 8, 9, 10	ICD-7	Before/After	Malignant
[18]								/Co-occurrence	
Ong et al.[19]	2014	UK	Cohort	219194	Caucasian	ICD-10	ICD-10	After	Malignant
Rugbjerg et al.[20]	2012	Denmark	Cohort	20343	Caucasian	ICD-8, ICD-10	ICD-10	After	Malignant
Fois et al.[22]	2010	UK	Cohort	4355	Caucasian	Hospitalization records	Medical records	Before/After	Benign/Malignant
Driver et al.[21]	2007	USA	Cohort	487	Caucasian	Self-report, Clinical diagnosis	Self-report, Medical records	After	Malignant
Oisen et al.[16]	2006	Denmark	Case-control	8090	Caucasian	ICD-8, ICD-10	ICD-7, ICD-O-1	Before	Malignant

ICD, international classification of diseases; ICD-O, international classification of diseases oncology. Brain tumor identification

*:“Before” refers to brain tumor identified before PD diagnosis, “After” represents brain tumor identified after PD diagnosis, “Co-occurrence” indicates brain tumor arising within 1 year before or after the index date for PD.

### Quality assessment of all included studies

Results of quality assessment by NOS scale for all the eight studies were in Table 2. The maximum score that could be achieved by a research is 9, while the scores of studies included in our analysis are ranged from 5–8, which suggests a moderate to good quality of these studies. All included studies in this meta-analysis fulfilled the Newcastle-Ottawa criteria.

**Table 2:** The quality of the included studies assessed by NOS.

Study	Selection				Comparability	Exposure			Scores
	Adequate definition of cases	Representativeness of cases	Selection of controls	Definition of controls	Control for important factor*	Ascertainment of exposure	Same method to ascertain for cases and controls	Non-response rate	
**Tang et al.**[15]	☆	☆	☆	☆	☆	☆	☆	-	7
**Lin et al.**[17]	☆	☆	☆	☆	☆☆	☆	☆	-	8
**Wirdefeldt et al.**[18]	☆	☆	☆	☆	☆	☆	☆	-	7
**Ong et al.**[19]	☆	☆	-	☆	☆	☆	☆	-	6
**Rugbjerg et al.**[20]	☆	☆	-	☆	☆	☆	☆	-	6
**Fois et al.**[22]	☆	-	-	☆	☆	☆	☆	-	5
**Driver et al.**[21]	☆	-	☆	☆	-	☆	☆	-	5
**Oisen et al.**[16]	☆	☆	-	☆	☆	☆	☆	-	6

*A maximum of two stars can be allotted in this category, one for Age, the other for other controlled factors.

### Overall association between brain tumor and PD

**Fig 2:**
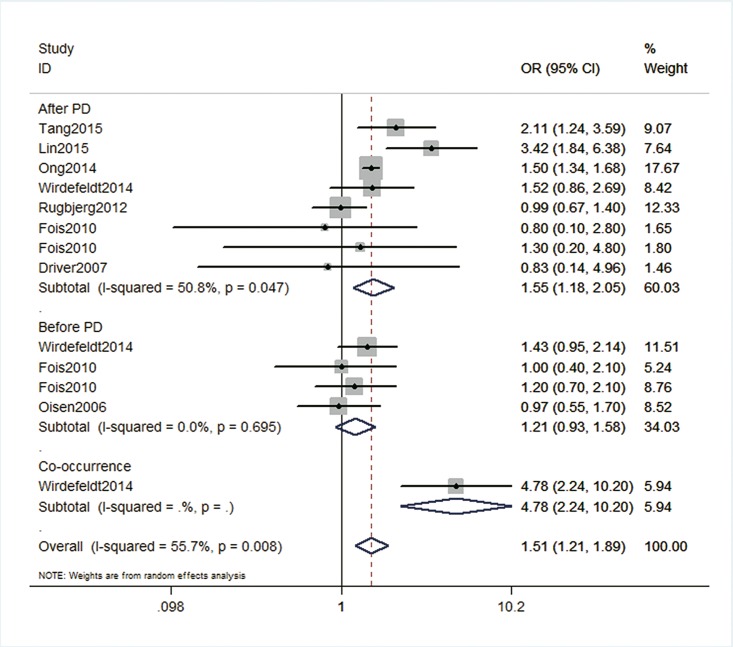
Forest plot of ORs for risk of brain tumor among PD patients. (OR 1.51, 95%CI 1.21–1.89, p<0.001, heterogeneity *I*^*2*^ = 55.7%, p = 0.008) and subgrouped by PD diagnosis time (brain tumor risk before PD, after PD or co-occurrence).

The results of the overall association between brain tumor and PD were shown in Fig 2. The pooled ORs for the relationship between brain tumor and PD across thirteen groups was 1.51 (95%CI 1.21–1.89, p<0.001, heterogeneity *I*^*2*^ = 55.7%, p = 0.008), indicating that PD carried a higher risk of brain tumor. Sensitivity analysis without the study of Ong et al.[19] gave similar results (OR 1.52, 95%CI 1.13–2.06, p = 0.006, heterogeneity *I*^*2*^ = 59.2%, p = 0.005), as well as omitting the study of Olsen et al.[16] (OR 1.58, 95%CI 1.25–2.00, p<0.001, heterogeneity *I*^*2*^ = 56%, p = 0.009). We also examined the source of this heterogeneity by excluding the study that reported the highest OR (4.78)[18]. Excluding this study from the analysis reduced the pooled OR to 1.41 (95% CI 1.17–1.71) and the degree of heterogeneity also reduced by nearly 20% (heterogeneity *I*^*2*^ = 38.1%, p = 0.087). In addition, a meta-regression was performed to explore the predefined possible source of heterogeneity. A lower degree of heterogeneity was also observed when publication year was included in the meta-regression (*I*^*2*^ = 38.3%) with a statistically significant effect on the pooled OR (p = 0.03).

### Occurrence of brain tumor following PD diagnosis

Eight groups examined the occurrence of brain tumor after PD diagnosis. Compared with individuals without PD, a consistent increased risk of brain tumor was observed among patients with PD across all studies (Fig 2), with a summary OR of 1.55 (95% CI 1.18–2.05, p = 0.002, heterogeneity *I*^*2*^ = 50.8%, p = 0.047). As mentioned above, after excluding the study that reported the highest OR (3.42)[17], the degree of heterogeneity reduced to 17.3% (p = 0.298) and an increased risk of brain tumor remained significance (OR 1.42, 95%CI 1.18–1.71, p = 0.012).

### Occurrence of brain tumor preceding PD diagnosis

Four groups examined the occurrence of brain tumor before PD diagnosis (Fig 2). The pooled OR for this subgroup was 1.21 (95%CI 0.93–1.58, p = 0.16) with no statistical heterogeneity (heterogeneity *I*^*2*^ = 0.0%, p = 0.695), indicating that no significant difference was found in risk of brain tumor preceding PD diagnosis.

### Co-occurrence of brain tumor and PD

Only one study examined the risk of cancers arising within 1 year before or after the index date for PD (Fig 2)[18]. Compared with expected prevalence in the age- and sex-matched populations in the nationwide Swedish health registries and the Swedish Multi-Generation Register, the prevalence of brain cancer in patients with PD was 4.78-fold higher (95% CI 2.24–10.20, p<0.001).

### Results of subgroup analysis

**Fig 3:**
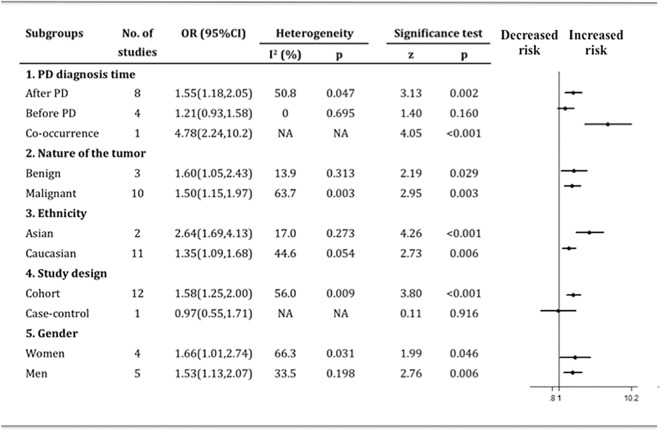
Pooled estimation on the risk of brain tumor in PD patients by subgroup analysis.

We carried out a panel of subgroup analyses on PD diagnosis time, the characteristics of the tumor, ethnicity, study design and gender (Fig 3). The influence of PD diagnosis time on the brain tumor risk was described above in detail. The nature of tumor (benign and malignant), ethnicity (Asian and Caucasian), and gender (women and men) did not have much influence on the occurrence of brain tumor risk among PD patients. In subgroup analysis of different study design, 12 groups were cohort study and only one was case-control study. In subgroup of cohort studies, the link between brain tumor and PD remained significant (OR 1.58, 95%CI 1.25–2.00, p <0.001), while no significance were found between brain tumor and PD in the case-control study of Oisen et al. (OR 0.97, 95%CI 0.55–1.71, p = 0.916)[16].

### Sensitivity analysis and publication bias

In sensitivity analysis, we recalculated the combined OR by omitting one study per iteration. Each study included in our meta-analysis showed no obvious influence on the effect size. Neither the funnel plot nor the publication bias was performed in any pooling analysis due to the included studies were less than ten.

## Discussion

To our knowledge, this was the first meta-analysis demonstrating the relationship between PD and brain tumor. The results presented here confirmed a positive association between PD occurrence and the risk of brain tumor. Subgroup analysis by different PD diagnosis time showed a consistent pattern of higher brain tumor occurrence among PD patients, while an inclined higher risk of PD without significant difference was found among patients with brain tumors. Besides, the positive association between PD occurrence and risk of brain tumor persists regardless of benign and malignant brain tumor, ethnicity and gender difference.

Our findings were supported by a previous study, which conducted a meta-analysis of cancer incidence related to central nervous system (CNS) disorders in more than 570,000 participants of 50 studies, and indicated that a lower risk of overall cancer was detected in PD patients, while PD was associated with a potential higher risk of brain cancer (Effect size = 1.21, 95% CI 0.95–1.52)[10]. Despite of the non-statistically significant result, a trend of increased co-occurrence of PD and brain cancer was identified, which was consistent with the result of our present meta-analysis.

In fact, previous studies have provided potential evidence that brain tumor and PD may share many pathophysiological processes including aging, mitochondrial dysfunction, oxidative stress, DNA damage, abnormal mitotic signaling, neuroinflammation and aberrant cell-cycle activation[7, 23]. These similarities between the pathology of brain tumor and PD suggested a positive epidemiologic association[24], which was verified in present meta-analysis. Besides, a-synuclein, as a major component of Lewy body and the pathological hallmark of PD[25], was also widely expressed in various brain tumors via its potential tumor stimulating effect[9, 26]. Furthermore, some of PD genes, including SNCA, PINK1 and ATP13A2, had already been found to be associated with brain tumors, indicating that the same mutations might lead to pathological changes of either PD or brain tumors[27–29]. Based on these previous researches and our current findings, more studies are needed to specify the potential biological links between PD and brain tumors, and to further develop new approaches to prevention and treatment of both diseases.

Nevertheless, attention should be paid to several other possible explanations for the different results classified by PD diagnosis time. The positive relationship between PD and brain tumors remains statistically significant in the settings of co-occurrence with PD diagnosis and after PD diagnosis, but not significant in the settings before PD diagnosis, which may result from ascertainment bias due to more frequent use of brain MRIs in the PD population[30]. That is to say, ascertainment bias due to increased medical surveillance and brain MRI scans could not be excluded in the analysis of PD and brain tumors, indicating that more studies addressing this issue, with longer follow-up and more accurate diagnosis, should be conducted in the settings before PD considering only four groups included in this field. Moreover, the drugs for PD treatment might play a role in higher risk of brain tumor occurrence after PD diagnosis, which remains unclear for the moment. Previous studies revealed that levodopa might have the impact on tumor generation by increasing the oxidative stress of cells[31], but the influence of levodopa on brain tumors remains unknown. Hence, future epidemiological studies, inspecting occurrence of brain tumor before PD diagnosis with larger sample size of brain tumors and strict inclusion criteria, are needed. At the mean time, the exact relationship between levodopa and brain tumor also warrants further investigation.

One strength of our research was that subgroup analysis was conducted to assess the potential effects of different factors and explore the possible source of heterogeneity. The present meta-analysis showed that gender differences, ethnicity differences and the characteristic of the tumor (benign or malignant) did not impact that much on the relationship between brain tumor and PD, and could not explain the main heterogeneity of these studies. While classified by PD diagnosis time, the degree of subgroup heterogeneity reduced to 0% among patients with brain tumor preceding PD diagnosis, indicating that PD diagnosis time might be one of the sources of heterogeneity. Previous studies have mentioned the possibility of secondary parkinsonism caused by brain tumors and the probable inclusion of vascular parkinsonism diagnosed by ICD-code[32, 33], which may interfere the exact association among patients with brain tumor preceding PD diagnosis, leading to the different results among subgroups classified by PD diagnosis time.

In addition, sensitivity analysis was also conducted to better explore the potential heterogeneity. After excluding the study of Oisen et al.[16] due to its different study design from others, the result of sensitivity analysis remained similar as original findings, further confirming the positive correlation between PD occurrence and risk of brain tumor. Besides, owing to the highest OR reported in the study of Wirdefeldt et al.[18] and hospital-based participants in the study of Ong et al.[19], these two individual studies were omitted separately by sensitivity analysis, which did not make much change of the result, presenting that the association between PD and brain tumor still remained statistically significant. Furthermore, our result of meta-regression presented that a lower degree of heterogeneity was also observed when publication year was included in the meta-regression, implying that the year of publication might be one source of heterogeneity in this meta-analysis.

However, we had to confess several limitations in this meta-analysis. First of all, most of these related analyses relied on small sample size for brain tumors. Besides, The majority of these studies were not originally designed to evaluate the relationship between brain tumors and PD; brain tumors were mostly assessed along with other tumors or medical conditions. Furthermore, data on risk factors, such as age, smokers and alcohol users, were limited from most of these included studies, which made it difficult to explore potential explanations. In spite of this limitation, we have tried our best to clarify the possible heterogeneity. It was suggested that the different PD diagnosis time and different year of publication, as well as variable factors adopted for calculating adjusted OR, might be seemingly plausible explanations for the statistical heterogeneity.

## Conclusion

Our meta-analysis suggests a moderate association of PD with a higher occurrence of brain tumor, while the influence of anti-parkinson drugs and ascertainment bias on our findings could not be excluded. More studies with larger sample size and more accurate diagnosis should be conducted in the future. Furthermore, it also warrants further investigation to examine the nature and mechanisms of this relationship and to develop probable common approaches to prevention and treatment of both diseases.

## Supporting Information

S1 File Study protocol. (PDF)

S2 File Search strategy. (PDF)

S1 Table PRISMA 2009 checklist. (DOC)
