# Supplementary material for: The Relationship between Parkinson Disease and Brain Tumor: A Meta-Analysis
Source: PLoS One. 2016 Oct 20;11(10):e0164388. doi: 10.1371/journal.pone.0164388 (PMC5072611; doi:10.1371/journal.pone.0164388)
Supplement: S1 File — (PDF) [file pone.0164388.s001.pdf]

S1 File. Study Protocol

The relationship between Parkinson disease and brain tumor: a systematic review protocol

Background

Parkinson disease (PD) is the second most common neurodegenerative disease, estimated to occur in approximately 1% of individuals older than 60 years, a number predicted to more than double by 2030 because of the aging population[1]. Resting tremor, rigidity, hypokinesia, and postural instability are the four cardinal motor symptoms of PD resulting from the loss of dopaminergic neurons in the substantia nigra pars compacta[2]. Accumulating epidemiological studies have reported associations between PD and cancers. In a recent meta-analysis, PD was inversely related to most types of cancers, which included both smoking-related and non-smoking-related cancers[3]. Besides, a positive relationship between PD and melanoma was also supported by a meta-analysis[4].

However, in recent studies investigating the association between PD and brain tumors, the results of which remain controversial[5]. Some reported a low occurrence of brain tumors in PD[6], while others indicated that brain tumors have higher morbidity among patients with PD[7]. In addition, a previous meta-analysis, which investigated cancer incidence related to central nervous system (CNS) disorders in more than 570,000 participants of 50 studies, indicated that PD was associated with a potential higher risk of brain cancer. It further revealed that a lower risk of overall cancer was detected in PD patients[8]. Despite of the non-statistically significant result, a trend of increased co-occurrence of PD and brain cancer was still identified.

Furthermore, previous studies have provided potential evidence that brain

tumor and PD may share many pathophysiological processes including aging, mitochondrial dysfunction, oxidative stress, DNA damage, abnormal mitotic signaling, inflammation and aberrant cell-cycle activation[9]. These similarities between the pathology of brain tumor and PD suggested a positive epidemiologic association[10]. Therefore, we decided to conduct a systematic review and meta-analysis to clarify the exact relationship between PD and brain tumors.

Objective

The aim of this study is to review the association between PD and brain tumors, and to assess the possible risk of brain tumor among patients with PD compared to non-PD participants in epidemiological longitudinal studies.

Methods

Inclusion and exclusion criteria

Eligible studies should meet the criteria presented below:

(1) Both PD and brain tumor were investigated in the study;

(2) Patients with parkinsonism should not be included;

(3) A measure of association (including an odds ratio [OR], relative risk [RR], or standardized incidence/event ratio [SIR/SER]), with 95% confidence intervals (CIs), should be reported for the association between PD and brain tumor in the study;

(4) Studies should be published in peer-reviewed journals without language restrictions;

(5) It should a longitudinal designed study.

Search strategy

Two investigators will independently search articles before 28 February, 2016 in four major electronic databases, including PubMed, Embase, ScienceDirect and CBM (China Biology Medicine Disc) by a full Boolean search logic method. We will perform this meta-analysis according to the recommendations of the Preferred Reporting Items for Systematic Reviews and Meta-analysis (PRISMA). Relevant text words and medical subject headings about PD, brain tumor and brain cancer will be included in the search strategy without restrictions.

Data extraction

Two investigators will independently rate the quality of the retrieved studies and extract data from eligible studies including first authors, publication year, sample size, mean age, regions, follow-up time in years, type of study design, PD diagnosis time and adjusted risk estimates and their 95% CIs.

Risk of bias assessment

The quality of cohort studies will be appraised according to the 9-star Newcastle-Ottawa Quality Assessment Scale. Disagreements will be solved by discussion or involvement a third reviewer if necessary.

Strategy for data synthesis

We will manage all data using STATA 12.0 (Stata Corporation, College Station, TX, USA) and set odds ratio (OR) at a 95% confidence interval (95% CI) to assess the association between PD and brain tumor. The involved measures (OR, RR, HR or SIR) will be treated equally under the assumption that both PD and brain tumors are rare. As lnOdds ratios (lnORs) will be considered to obey normal distribution,

lnORs and the corresponding ln lower limits (lnLLs) and ln upper limits (lnULs) will be used as data points in pooling analysis.

Analysis of subgroups or subsets

Subgroup analysis will be conducted and subgrouped by different study design, PD diagnosis time, gender difference, characteristics of the tumor and ethnicity.

References

1. Kalia LV, Lang AE. Parkinson's disease. The Lancet. 2015;386(9996):896-912.

2. Postuma RB, Berg D, Stern M, Poewe W, Olanow CW, Oertel W, et al. MDS clinical diagnostic criteria for Parkinson's disease. Movement disorders : official journal of the Movement Disorder Society. 2015;30(12):1591-601.

3. Bajaj A, Driver JA, Schernhammer ES. Parkinson's disease and cancer risk: a systematic review and meta-analysis. Cancer causes & control : CCC. 2010;21(5):697-707.

4. Liu R, Gao X, Lu Y, Chen H. Meta-analysis of the relationship between Parkinson disease and melanoma. Neurology. 2011;76(23):2002-9.

5. Feng DD, Cai W, Chen X. The associations between Parkinson's disease and cancer: the plot thickens. Translational neurodegeneration. 2015;4:20.

6. Olsen JH, Friis S, Frederiksen K. Malignant melanoma and other types of cancer preceding Parkinson disease. Epidemiology. 2006;17(5):582-7.

7. Tang CF, Lu MK, Muo CH, Tsai CH, Kao CH. Increased risk of brain tumor in patients with Parkinson's disease: a nationwide cohort study in Taiwan. Acta neurologica Scandinavica. 2015.

8. Catala-Lopez F, Suarez-Pinilla M, Suarez-Pinilla P, Valderas JM, Gomez-Beneyto M, Martinez S, et al. Inverse and direct cancer comorbidity in people with central nervous system disorders: a meta-analysis of cancer

incidence in 577,013 participants of 50 observational studies. Psychotherapy and psychosomatics. 2014;83(2):89-105. doi: 10.1159/000356498. PubMed PMID: 24458030.

9. Tansey MG, Goldberg MS. Neuroinflammation in Parkinson's disease: its role in neuronal death and implications for therapeutic intervention. Neurobiology of disease. 2010;37(3):510-8. doi: 10.1016/j.nbd.2009.11.004. PubMed PMID: 19913097; PubMed Central PMCID: PMC2823829.

10. Driver JA. Inverse association between cancer and neurodegenerative disease: review of the epidemiologic and biological evidence. Biogerontology. 2014;15(6):547-57. doi: 10.1007/s10522-014-9523-2. PubMed PMID: 25113739.
