# Supplementary material for: The Relationship between Parkinson Disease and Brain Tumor: A Meta-Analysis
Source: PLoS One. 2016 Oct 20;11(10):e0164388. doi: 10.1371/journal.pone.0164388 (PMC5072611; doi:10.1371/journal.pone.0164388)
Supplement: S2 File — (PDF) [file pone.0164388.s002.pdf]

**S2 File. Search strategy**

Relevant studies, published before February 28, 2016, were identified through electronic searches using PubMed, Embase, ScienceDirect and CBM (China Biology Medicine Disc) databases. Electronic searches were supplemented by scanning reference lists of articles identified for all relevant studies, by hand searching of relevant journals and by correspondence with study investigators. The computer-based searches combined search terms related to Parkinson disease and brain tumors. Below is the detailed search strategy for PubMed.

**Table 2. Search strategy for PubMed.**

| **To locate PD** |
|---|
| 1. Parkinson Disease [Mesh] |
| 2. Parkinson Disease [tw] |
| 3. Parkinson's Disease [tw] |
| 4. Parkinsons Disease [tw] |
| 5. Or/1-4 |
| **To locate brain tumors** |
| 6. Brain neoplasms [Mesh] |
| 7. Glioma [Mesh] |
| 8. Glioma* [tw] |
| 9. Glial cell tumor* [tw] |
| 10. Brain neoplasm* [tw] |
| 11. Brain tumor* [tw] |
| 12. Brain cancer* [tw] |
| 13. Intracranial neoplasm* [tw] |
| 14. Or/6-13 |
| 15. 5 and 14 |
